# Dynamic cholinergic signaling differentially desynchronizes cortical microcircuits dependent on modulation rate and network connectivity

**DOI:** 10.1371/journal.pcbi.1013252

**Published:** 2026-04-10

**Authors:** Sibi Pandian, Scott Rich

**Affiliations:** 1 Department of Physiology and Neurobiology, University of Connecticut, Storrs, Connecticut, United States of America; 2 Department of Biomedical Engineering, University of Connecticut, Storrs, Connecticut, United States of America; 3 Department of Mathematics and Institute for the Brain and Cognitive Sciences, University of Connecticut, Storrs, Connecticut, United States of America; Indiana University Indianapolis and Indiana University School of Medicine, UNITED STATES OF AMERICA

## Abstract

Acetylcholine (ACh) affects both the intrinsic properties of individual neurons and the oscillatory tendencies of cortical microcircuits by modulating the muscarinic-receptor gated m-current. ACh concentrations have historically been assumed to vary exclusively over long (supra-second) neuromodulatory timescales, conventionally simplified *in silico* as a set and constant modulatory tone. However, contemporary experimental studies show cortical ACh concentrations change over sub-second timescales associated with cognitive tasks including attention and sensorimotor coordination. More realistic models reflecting dynamic, sub-second fluctuations in cholinergic tone have yet to be computationally studied. Using a new implementation of a time-varying cholinergic signal in computational excitatory-inhibitory (E-I) spiking neuronal networks, we here delineate how the interaction between dynamic cholinergic modulation and network connectivity influences these systems’ oscillatory tendencies. Synchrony in networks with dominant inter-connectivity (strong E-to-I and I-to-E synapses) is largely unaffected by time-varying cholinergic modulation. In contrast, networks with dominant intra-connectivity (strong E-to-E and I-to-I synapses) desynchronize with increasing cholinergic tone in manners diverging from the predictions of analogous systems with constant ACh levels. The rate and mechanism of this desynchronization is highly sensitive to the modulation’s time course and the E-I connectivity strength. This suggests that traditional *in silico* simplifications of the temporal profile of cholinergic activity may obscure sub-second neuromodulatory effects, which may be particularly relevant to contemporary efforts to optimize neurostimulation therapies influencing cholinergic pathways.

## Author summary

Acetylcholine (ACh) is a chemical messenger that alters the intrinsic properties of neurons and in turn regulates brain activity related to cognitive functions such as sensory processing, memory formation, and attention. Although mounting experimental evidence shows that ACh concentrations in the cortex change dynamically at sub-second timescales, computational studies traditionally consider ACh concentrations exclusively as constant across time. This motivated our new *in silico* model of a neuronal microcircuit where cellular properties are affected by ACh concentrations varying over these fast timescales. The oscillatory dynamics of this microcircuit differ in key fashions from similar systems with constant ACh levels, including critical dependence upon the synaptic connectivity of the modeled brain region. These results describe novel mechanisms by which ACh controls microcircuit activity overlooked in conventional models, an understanding of potential importance to the refinement of neurostimulation therapies acting through transient alterations to cholinergic activity.

## Introduction

Acetylcholine (ACh) is traditionally assumed to modulate the dynamics of neuronal microcircuits over slow (at least supra-second) timescales, facilitating cognitive processes such as attention, sensory processing, and memory formation [1]. Indeed, high levels of ACh are present during wakefulness and low levels during sleep, a phenomenon potentially contributing to the desynchronization and decorrelation of cortical activity during awakening [2,3]. Meanwhile, deterioration of cholinergic projections from the basal forebrain to the cortex correlates with the onset of Alzheimer’s disease and cognitive decline [4]. However, recent technological advancements improving the temporal resolution of ACh measurements [5,6] challenge the assumption that cholinergic signaling in the cortex is exclusively slow and diffusive [7]. *In vivo* studies identify cholinergic transients, initiating and peaking on the order of hundreds of milliseconds, in response to relevant stimuli [8]. These signals are associated with perceptual tasks demanding immediate response [1,4,7] such as cue-detection [9] and foot shock-reactions [10].

Among its many effects, cholinergic modulation changes neurons’ cellular excitability as ACh binds to muscarinic receptors. The current mediated by the M1 receptor [11] is of particular interest and is colloquially referred to as the “m-current.” Modulation of the m-current affects both intrinsic cellular properties and microcircuit activity patterns [7]. Contemporary experimental studies implicate this neuromodulatory pathway as a likely candidate for mediating the faster effects of cholinergic modulation [12]. This literature includes the finding that vagal nerve stimulation (VNS) causes rapid disruption of cortical oscillations [13] potentially mediated by muscarinic activity [14].

Computational studies of conductance-based neuron models with an m-current have proposed mechanisms explaining the cellular and microcircuit-level effects of tonic changes in cholinergic tone [15–18]. In such models, ACh’s modulatory effects are conventionally represented as decreasing the magnitude of a model m-current [19], accounting primarily for current through M1 receptors as well as some effects of other muscarinic-receptor gated slow potassium currents [20,21]. Blocking this current reproduces the increased excitability, decreased spike frequency adaptation, and altered phase response curves (PRCs)—a measurement of how perturbations to a neuron at different stages in its oscillatory cycle temporally shift subsequent spikes [22,23]—observed experimentally as cholinergic tone increases [19,24].

These studies most commonly focus on the effects of cholinergic modulation on excitatory-inhibitory (E-I) networks, which consist of interconnected excitatory and inhibitory neuron populations. E-I networks are a ubiquitous *in silico* model of simplified cortical microcircuits [25,26] used historically to study microcircuit oscillations [27], including the derivation of the seminal Pyramidal Interneuron Network Gamma (PING) mechanism [28]. Recent studies have characterized functional effects of cholinergic modulation in these systems [29–33] and identified dichotomous effects in dominant inter-connectivity (strong E-to-I and I-to-E synapses) versus dominant intra-connectivity (strong E-to-E and I-to-I synapses) networks [17]. However, these studies uniformly overlook the potential influence of cholinergic modulation varying continuously over sub-second timescales.

To address this gap, we here investigate how a dynamic model of cholinergic modulation of the m-current affects network synchrony (and associated oscillatory dynamics) in E-I networks, identifying unique effects of such signaling and proposing mechanistic explanations for this activity. We compared the effects of tonic and dynamic modulation across differing connectivity schemes and varying rates of modulation. Our explorations reveal that dynamic cholinergic modulation preferentially affects dominant intra-connectivity networks and that oscillatory activity in these systems can be disrupted via two distinct mechanisms. Surprisingly, relatively low magnitude changes in E-I synaptic strength trigger a switch between these desynchronization mechanisms. These *in silico* results are the first insights into the interacting effects of network connectivity and sub-second changes in cholinergic tone on cortical microcircuit dynamics.

## Materials and methods

### Neuron model

We model all excitatory and inhibitory cells in our E-I network with a Hodgkin-Huxley formalism containing a conductance-based m-current. While this model was originally designed as a cortical pyramidal neuron model [19,23], it has been used unaltered as a viable model of inhibitory interneurons [15–17]. Indeed, when the m-current is fully blockaded the firing properties of this model phenomenologically approximate those of fast-spiking, parvalbumin-positive interneurons [34]. Additionally, there is evidence of both the m-current and spike-frequency adaptation (a paramount effect of the m-current model implemented here) among the diversity of cortical interneurons [21,35]. The equations dictating this model are as follows:


$$\begin{array}{cc} \frac{dV}{dt} & =-g_{Na}m_{\infty }^{3}h(V-E_{Na})-g_{K_{d}}n^{4}(V-E_{K_{d}})-g_{K_{s}}z(V-E_{K_{s}})\\ & -g_{L}(V-E_{L})+I_{app}-I_{syn}\; \end{array}$$ (1)

$$\frac{dX}{dt}=\frac{X_{\infty }(V)-X}{\tau_{X}(V)}forX=h,n,z$$ (2)

$$m_{\infty }(V)=\frac{1}{1+e^{(-V-30)/9.5}}$$ (3)

$$h_{\infty }(V)=\frac{1}{1+e^{(V+53)/7.0}}$$ (4)

$$n_{\infty }(V)=\frac{1}{1+e^{(-V-30)/10}}$$ (5)

$$z_{\infty }(V)=\frac{1}{1+e^{(-V-39)/5}}$$ (6)

$$\tau_{h}(V)=0.37+\frac{2.78}{1+e^{(V+40.5)/6}}$$ (7)

$$\tau_{n}(V)=0.37+\frac{1.85}{1+e^{(V+27)/15}}$$ (8)

$$\tau_{z}(V)=75$$ (9)

*V* represents the voltage of the neuron in mV, while *m*, *h*, *n*, and *z* represent the unitless gating variables of the ionic current conductances. $I_{app}$ and $I_{syn}$ signify the external applied current and the synaptic current (defined in Network Structure) respectively in $\mu \text{A}/{\text{cm}}^{2}$. $E_{Na}$, $E_{K_{d}}$, $E_{K_{s}}$, and $E_{L}$ are the reversal potentials, and $g_{Na}$, $g_{K_{d}}$, $g_{K_{s}}$, and $g_{L}$ the maximum conductances of the sodium channel, delayed rectifier potassium channel, slow m-type potassium channel, and leak-channel respectively. We use $E_{Na}$ = 55 mV, $E_{K_{d}}$ = $E_{K_{s}}$ = -90 mV, $E_{L}$ = -60 mV, $g_{Na}$ = 24 mS/cm^2^, $g_{K_{d}}$ = 3 mS/cm^2^, and $g_{L}$ = 0.02 mS/cm^2^.

We model dynamic cholinergic modulation by subjecting $g_{k_{s}}$ to a linear decline from 1.5 to 0 mS/cm^2^ for all cells, after allowing dynamics to initially stabilize at the maximal $g_{k_{s}}$ for 1000 ms. This range of $g_{k_{s}}$ values allows us to replicate experimentally recorded ACh-induced changes in F-I curves and PRCs [19].


$$\begin{array}{c} g_{ks}=\left\{ \begin{array}{cc} 1.5 & t<1000\\ 1.5-rt & 1000\leq t<1000+\frac{1.5}{r}\\ 0 & t\geq 1000+\frac{1.5}{r} \end{array}\right.\; \end{array}$$

Here, *t* refers to the time in ms and *r* refers to the rate of linear decline in (mS/cm^2^)/ms. We use various rates of modulation through the study, with the fastest rate of modulation allowing $g_{k_{s}}$ to drop from 1.5 to 0 mS/cm^2^ in 1 second and the slowest allowing the same in 8 seconds. These timescales are selected to approximate the experimentally recorded kinetics of muscarinic ACh binding [36,37] and unbinding [38], which occur over hundreds of milliseconds. Additionally, we study scenarios where we permanently set the inhibitory cells’ $g_{k_{s}}$ to 0 mS/cm^2^, only carrying out modulation on excitatory cells.

### Network structure

Our E-I network consists of 1000 neurons, 800 excitatory and 200 inhibitory, following the ubiquitous 4:1 ratio of E:I neurons used in the computational study of such microcircuits [16,17,39,40]. We establish inter-connectivity by allowing each excitatory cell a 50% chance to synapse onto each inhibitory cell, and each inhibitory cell likewise a 50% chance to synapse onto each excitatory cell. Additionally, we establish intra-connectivity by allowing each cell in the excitatory and inhibitory populations to have a 30% percent chance of synapsing onto other cells within their own population. These choices are as used in previous theoretically-focused work, facilitating direct comparison to analogous results with tonic cholinergic modulation [17]. We modeled synaptic connections using a conductance-based double-exponential profile of the form


$$I_{syn}(t)=g_{syn}(V-E_{syn})\left(\sum_{s_{i}}e^{-(t-s_{i})/\tau_{d}}-e^{-(t-s_{i})/\tau_{r}}\right)$$ (10)

where $g_{syn}$ is the maximum synaptic conductance, *V* is the membrane voltage of the post-synaptic neuron, $E_{syn}$ is the reversal potential of the synaptic current, $s_{i}$ are the times of all presynaptic spikes occurring before the current time t in ms, and $\tau_{d}$ and $\tau_{r}$ are the synaptic decay and synaptic rise constants respectively. We set $E_{syn}$ to -75 mV for inhibitory synapses and to 0 mV for excitatory synapses. $\tau_{r}$ is set to 0.2 ms for all synapses, while $\tau_{d}$ is 3.0 ms for excitatory synapses (approximating fast AMPA-mediated signaling [41]) and 5.5 ms for inhibitory synapses (approximating fast GABA_A_ signaling [42]). To vary network connectivity, we control the strengths of the E-E, E-I, I-E, I-I synapses by individually altering the synaptic weights ($g_{syn}$) for each type of synapse. We model dominant inter-connectivity with synaptic weight parameters as E-I, I-E = 0.00175 mS/cm^2^, E-E = 0.0000625 mS/cm^2^, and I-I = 0.00025 mS/cm^2^, which promotes network oscillations with PING-like dynamics. For dominant intra-connectivity, we use E-I, I-E = 0.00025 mS/cm^2^, E-E = 0.000125 mS/cm^2^, and I-I = 0.0005 mS/cm^2^, which promotes network oscillations controlled by PRC-driven effects [17]. We note that these synaptic time constants and the equivalent E-I and I-E synaptic weights mirror previous work [17] rather than a specific brain region or cell type, facilitating the conclusion that alterations in network activity are driven primarily by dynamic rather than tonic cholinergic modulation and not a confounding alteration.

To introduce cellular heterogeneity, we choose $I_{app}$ for each excitatory cell such that, in isolation, the cell would fire at a randomly selected frequency within the uniform distribution (45 Hz, 55 Hz). Adding heterogeneity to the external inputs is common in the study of PING rhythmicity [28] to approximate heterogeneous neuronal excitability and inputs *in vivo*. As $g_{k_{s}}$ declines and cellular properties are changed, we concurrently modulate $I_{app}$ for every 0.01 mS/cm^2^ change in $g_{k_{s}}$ to preserve the originally selected isolated firing frequencies of the excitatory cells. Meanwhile, the inhibitory cells’ firing is driven largely by the excitatory signaling. $I_{app}$ for the inhibitory population is set such that the cells are brought closer to their threshold for firing, but does not by itself permit them to spike. Here too, we incorporate cellular heterogeneity and select $I_{app}$ for each cell from a (0.95 $I_{A}$, 1.05 $I_{A}$) uniform distribution, where $I_{A}$ is the average current. We similarly modulate $I_{A}$ for every 0.01 mS/cm^2^ change in $g_{k_{s}}$ to keep the inhibitory cells near the threshold of firing. At maximal $g_{k_{s}}=1.5$ mS/cm^2^, $I_{A}$ is 1.0 $\mu \text{A}/{\text{cm}}^{2}$, and at minimal $g_{k_{s}}=0$ mS/cm^2^, $I_{A}$ is -0.14 $\mu \text{A}/{\text{cm}}^{2}$.

### Measures

To characterize network synchrony we employ a quantification dubbed the “Synchrony Measure” in previous work [15–17], a modified version of the measure developed in [43,44]. This measures the degree of spike coincidence in a given neuron population for a specified time window. We define a voltage approximation for each neuron, $V_{i}(t)$, by convolving a Gaussian function with a series containing each neuron’s spike times over the window of interest. We note that this approach ensures our measure is influenced only by spiking activity, rather than subthreshold voltage fluctuations. We obtain the population-averaged voltage approximation $V(t)=\frac{1}{N}\sum_{i=1}^{N}V_{i}(t)$, where N is the number of cells. Using $V_{i}(t)$ and *V*(*t*), we calculate the variance of the population voltage σ and variance of the individual neurons’ voltages $\sigma_{i}$ as


$$\sigma =\langle V(t{)}^{2}\rangle -\langle V(t)\rangle^{2}$$ (11)

$$\sigma_{i}=\langle V_{i}(t{)}^{2}\rangle -\langle V_{i}(t)\rangle^{2}$$ (12)

where ⟨⟩ denotes averaging over the time window. The Synchrony Measure S is then defined as


$$S=\frac{\sigma }{\frac{1}{N}\sum_{i=1}^{N}\sigma_{i}}$$ (13)

S assumes values from 0 to 1, with S = 0 denoting a completely asynchronous population, increasing values demarcating more coincident network spiking and synchrony, and S = 1 denoting a perfectly synchronized population. We use rolling time windows without overlap to measure synchrony around different $g_{k_{s}}$ ranges, with the first window starting 1000 ms into the simulation when $g_{k_{s}}$ decline activates. For the simulations shown in Figs 5–8, $g_{k_{s}}$ decline is set to 0.67 (mS/cm^2^)/s and the window lengths are set to 150 ms. For the simulations shown in Fig 4, the window lengths are set to either 50, 100, 200, or 400 ms, based on the four rates of $g_{k_{s}}$ decline—networks with faster decline use smaller window lengths. Measuring the level of synchronous spiking in moving time windows in this analog fashion allows for quantification of the rate at which network desynchronization occurs. We also quantify synchrony for networks with tonic $g_{k_{s}}$ in Fig 4; for these cases, we use the last 1000 ms of a 2000 ms simulation as the window.

### Simulations

Code for all simulations is written in Python. We initialize each simulation with random initial conditions for each cell in the E-I network. *V* is selected from the uniform distribution (-62 mV, 22 mV), *n* and *h* from (0.2, 0.8), and *z* from (0.15, 0.25). Model equations are evaluated numerically using the fourth-order Runge-Kutta technique. We include synaptic current only after the first 100 ms of the simulation to allow initial transients to decay.

All raster plots are organized such that cells with higher $I_{app}$ have a lower neuron index and are placed closer to the bottom of the plots and vice versa. This allows for clearer visualization of the factors impacting temporal organization within individual bursts. For all figures with measures, we plot the average of the measures across ten independent simulations. In all cases, we only compute measures based on network activity after the first 1000 ms, again to allow dynamics to stabilize.

## Results

### Synaptic connectivity strengths shape the response to dynamic cholinergic modulation

**Fig 1:**
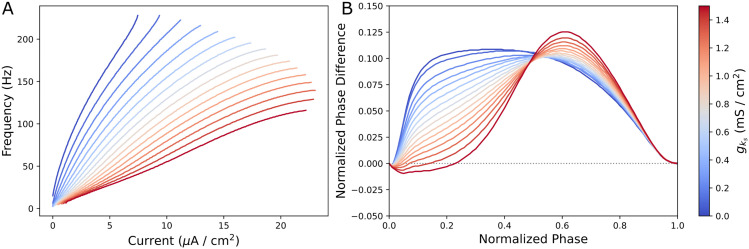
Changing intrinsic neuronal properties as a function of varying $g_{k_{s}}$. **A-B**: Frequency-Current (F-I) curves (Panel **A**) and phase response curves (PRCs, Panel **B**) for neurons with differing $g_{k_{s}}$. At $g_{k_{s}}$ = 1.5 mS/cm^2^, the m-current is maximally active and at $g_{k_{s}}$ = 0 mS/cm^2^, the m-current is fully blocked by ACh. Panel **A** shows that neurons with lower $g_{k_{s}}$ have steeper F-I curves, exhibiting higher excitability, and are capable of firing with lower current input. Panel **B** shows that neurons with higher $g_{k_{s}}$ have a negative, phase delay region in response to an excitatory stimulus early in the neuron’s firing cycle. Cholinergic modulation induces a continuous shift in F-I curve and PRC properties.

The cellular effects of varying levels of $g_{k_{s}}$ are illustrated in Fig 1. The extreme values of $g_{k_{s}}=1.5$ and 0 mS/cm^2^ have been conventionally used to reflect static “low” and “high” cholinergic tone in previous studies [17]. Closure of the m-current, realized via decreasing $g_{k_{s}}$, increases neuronal excitability (Fig 1A) and reduces the width of the phase delay region of the PRC (the window immediately following a spike in which an excitatory stimulus delays the subsequent action potential; Fig 1B). This phase delay region promotes synchrony in populations of recurrently connected excitatory neurons [45], and the specific PRC differences in this model with varying $g_{k_{s}}$ have been shown to drive differing network synchronization patterns [15,46].

**Fig 2:**
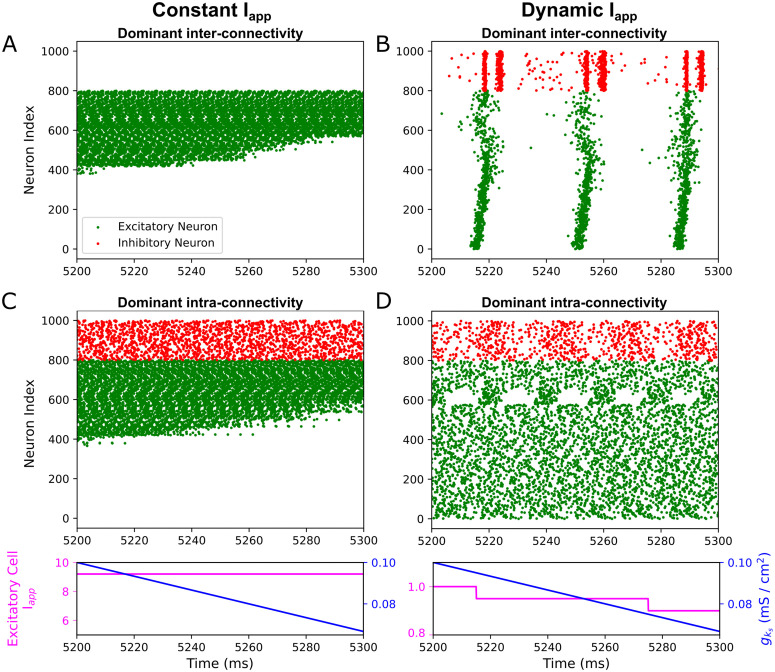
Dynamic m-current blockade in E-I networks leads to depolarization block in the absence of concurrent external current modulation. **A-D**: Raster plots showing 100 ms periods of activity in E-I networks. Panels **A** and **B** are dominant inter-connectivity networks and Panels **C** and **D** are dominant intra-connectivity networks (synaptic weight parameters as specified in the Materials and Methods). Linear $g_{k_{s}}$ downregulation is carried out in all networks at rate 0.33 (mS/cm^2^)/s. $g_{k_{s}}$ level is displayed below the raster plots, along with the average external current to an excitatory neuron in $\mu \text{A}/{\text{cm}}^{2}$, which is constant in Panels **A** and **C** and changing in Panels **B** and **D** to keep the firing frequency of an isolated average neuron approximately constant. Depolarization block occurs in response to declining $g_{k_{s}}$ by default, with all excitatory neurons ceasing to fire (Panels **A** and **C**). However, when we account for higher neuron excitability induced by $g_{k_{s}}$ decrease by accordingly downregulating the external current, depolarization block does not occur (Panels **C** and **D**), preventing the biophysically unlikely phenomenon of cholinergic modulation inducing full depolarization block.

We first implemented dynamic cholinergic modulation, as realized in $g_{k_{s}}$ values varying linearly over time (detailed in the Materials and Methods), on E-I networks with either dominant inter-connectivity (E-I and I-E synapses much stronger than E-E and I-I synapses) or dominant intra-connectivity (E-E and I-I synapses much stronger than E-I and I-E synapses). As $g_{k_{s}}$ linearly decreases in these exemplar simulations (Fig 2A and 2C), we note that the excitatory cells are driven to fire at increasingly higher frequencies until they all cease to fire. Examining the voltage traces of the neurons confirmed that the cessation of firing was due to depolarization block. In Fig 2A, mean excitatory cell firing frequency increases from 56.9 Hz in the first 100 ms window after $g_{k_{s}}$ modulation begins to 208.7 Hz in the 100 ms window immediately preceding the first excitatory neuron entering depolarization block at 5032 ms. All inhibitory neurons also cease to fire in Fig 2A, due to the heightened E-I connectivity providing excessive synaptic input to the inhibitory neurons. Meanwhile, in Fig 2C, the increase is from 50.0 Hz to 204.9 Hz over the same intervals, with the first excitatory cell’s depolarization block at 5026 ms.

Termination of activity across all cells in a cortical microcircuit is a physiologically unrealistic response to cholinergic modulation. This *in silico* phenomenon arises primarily from changes to the F-I curve as $g_{k_{s}}$ decreases (Fig 1A), increasing cellular excitability in a fashion that drowns out other effects of cholinergic modulation. To characterize effects obscured by depolarization block in this first experiment, we introduced simultaneous modulation of the neurons’ $I_{app}$ while preserving the system’s heterogeneity (detailed in the Materials and Methods). This strategy controls for large changes in the baseline firing rate of isolated neurons while capturing more realistic changes in network firing frequency driven by the F-I curve’s gain. For example, in the network of Fig 2D, we observe a steady but moderate increase in the excitatory population’s mean firing frequency as $g_{k_{s}}$ declines (50.0 Hz during the first 100 ms after $g_{k_{s}}$ modulation starts and 56.7 Hz in the final 100 ms as $g_{k_{s}}$ modulation ends). This technique phenomenologically accounts for the host of other concurrent modulatory effects that might homeostatically maintain a cortical microcircuit’s firing activity as the m-current is blocked, some of which are described further in the Discussion.

These modifications successfully prevent depolarization block in the example microcircuits (Fig 2B and 2D). Observing the stable end behaviors reached by both networks (i.e., long after $g_{k_{s}}$ reached 0 mS/cm^2^), we found that only the dominant inter-connectivity network retained organized firing (Synchrony Measure of 0.53 over a 500ms window after modulation ends) and that the dominant intra-connectivity network transitioned into asynchronous firing (Synchrony Measure of 0.02 over the same window). These end behaviors correspond with findings from networks with tonic cholinergic tone [17]: networks with dominant intra-connectivity fire asynchronously at low ACh concentrations—as they depend upon the phase delay region of the excitatory cells’ PRCs for synchronization—while networks with dominant inter-connectivity synchronize independent of ACh levels. These results are affirmative support that a network whose $g_{k_{s}}$ decreases dynamically will match the dynamics of a network with tonically low $g_{k_{s}}$ if the system is allowed to equilibrate. For the remainder of this manuscript, we will continue to modulate $I_{app}$ in the manner discussed here.

**Fig 3:**
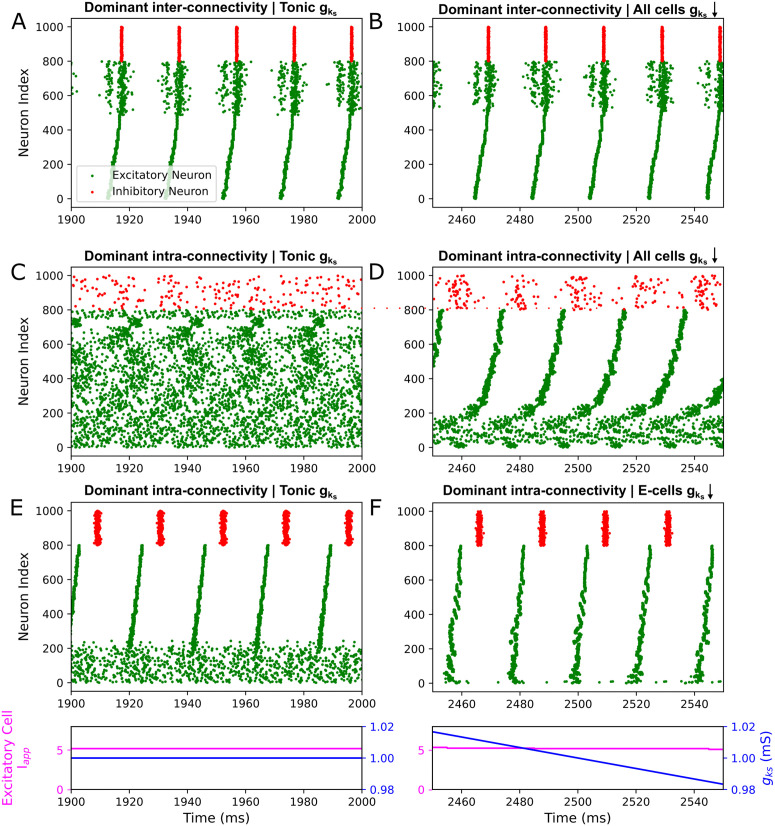
Differences in network activity caused by tonic versus dynamic cholinergic modulation arise preferentially in networks with dominant intra-connectivity. **A-F**: Raster plots showing 100 ms of activity in E-I networks. A static $g_{k_{s}}=1.0$ mS/cm^2^ is used in Panels **A**, **C**, and **E**. In contrast, $g_{k_{s}}$ declines linearly at 0.33 (mS/cm^2^)/s in Panels **B**, **D**, and **F**, with the raster plot displaying a window centered around the time at which $g_{k_{s}}=1.0$ mS/cm^2^. Panels **A** and **B** have dominant inter-connectivity and Panels **C-F** have dominant intra-connectivity, with parameters for synaptic weights as specified in the Materials and Methods. Panels **E** and **F** have $g_{k_{s}}$ permanently set to 0 mS/cm^2^ for inhibitory cells. Dominant inter-connectivity networks show consistent organization of excitatory and inhibitory cells with dynamic (Panel **B**) or tonic (Panel **A**) $g_{k_{s}}$, with the Synchrony Measure over the 100 ms window being 0.58 and 0.57 respectively. In contrast, dominant intra-connectivity networks display divergent patterning of the cells: Panels **D** and **F** are more synchronized (Synchrony Measures: 0.30, 0.68) compared to Panels **C** and **E** (0.02, 0.52). The dominant intra-connectivity networks’ ability to fire synchronously is dependent upon cellular properties, which are influenced by the m-current, explaining their tendency for altered firing patterns from changing $g_{k_{s}}$.

We next asked whether the firing patterns exhibited in networks with dynamically changing $g_{k_{s}}$ but *without* time to equilibrate, would match the equilibrium dynamics of networks with an analogous constant $g_{k_{s}}$ value. To do so, we compared the stable end behavior reached by networks simulated with an intermediate $g_{ks}=1.0$ mS/cm^2^ (Fig 3A, 3C and 3E) with the transient behavior of networks with time-varying $g_{k_{s}}$ at a time window centered around 1.0 mS/cm^2^ (Fig 3B, 3D and 3F).

**Fig 4:**
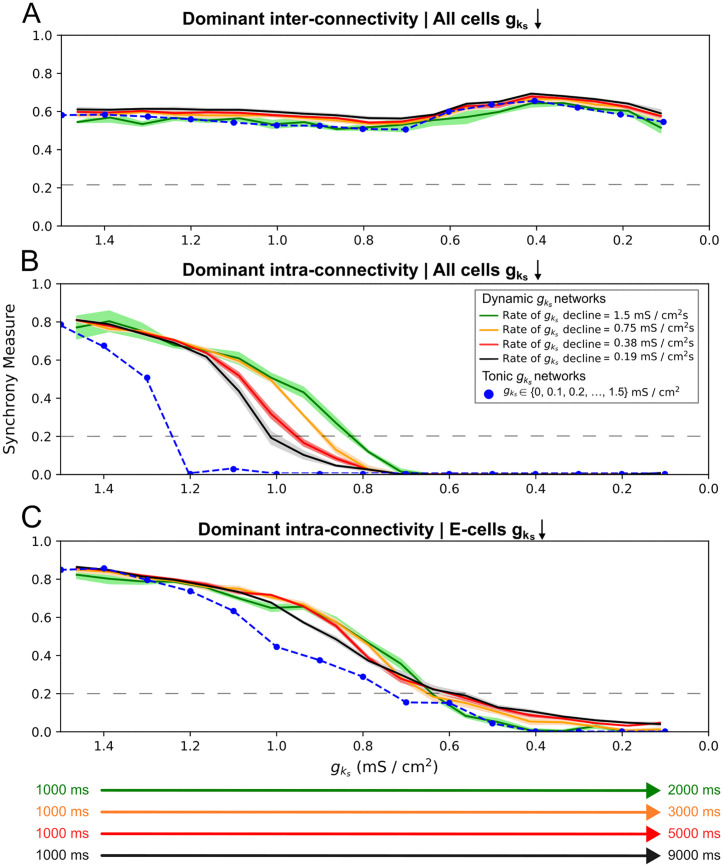
Rate of m-current blockade affects the relationship between desynchronization and $g_{k_{s}}$ only in dominant intra-connectivity networks. **A-C**: Synchrony measure of excitatory cells plotted over $g_{k_{s}}$ for networks with varying rates of $g_{k_{s}}$ modulation and for networks with tonic $g_{k_{s}}$. Panel **A** includes networks with dominant inter-connectivity and Panels **B** and **C** include networks with dominant intra-connectivity, with parameters for synaptic weights as specified in the Materials and Methods. Panel **C** networks have $g_{k_{s}}$ permanently set to 0 mS/cm^2^ for inhibitory cells. Color-coded arrows show the timecourse of $g_{k_{s}}$ modulation from 1.5 to 0 mS/cm^2^ for each network. All measures plotted from the dynamic networks are the mean based on 10 independent simulations, with ± standard deviation shading. Values for tonic networks are means of the Synchrony Measure over the last 1000 ms from 10 independent 2000 ms simulations. Network dynamics are classified as desynchronized when the Synchrony Measure is below 0.2 (grey-dashed lines in each panel). In Panel **A**, no substantial changes in synchrony are recorded in response to time-varying $g_{k_{s}}$. Conversely, in Panels **B** and **C** desynchronization occurs, with slower rates of $g_{k_{s}}$ decline generally resulting in desynchronization starting at a a higher $g_{k_{s}}$ value. These observations indicate that strong inter-connectivity allows for synchronous network activity that is robust to cholinergic modulation regardless of its timecourse; however, for networks with dominant intra-connectivity, cholinergic modulation and its timescale affect the propensity for synchronous network activity over time.

In the dominant inter-connectivity networks, tonic (Fig 3A) versus dynamic (Fig 3B) cholinergic modulation induces no notable differences in the firing patterns. In contrast, there are conspicuous differences in the qualitative degree of network synchrony in dominant intra-connectivity networks with tonic (Fig 3C and 3E) and dynamic (Fig 3D and 3F) cholinergic modulation. The excitatory neurons in the examples with dynamic cholinergic modulation illustrated in Fig 3D and 3F (the former models joint cholinergic modulation of the excitatory and inhibitory neurons, the latter cholinergic modulation only in excitatory neurons) form distinctly more organized bursts in comparison to the corresponding examples in Fig 3C and 3E in which $g_{k_{s}}$ is constant. Although we only showcase results from $g_{k_{s}}$ 1.0 mS/cm^2^, similar relationships between tonic and dynamic models were found with other intermediate $g_{k_{s}}$ values during our investigations (see discussion of Fig 4 below).

This preliminary investigation suggests that unique responses to time-varying ACh levels arise primarily in networks with dominant intra-connectivity. In networks with dominant inter-connectivity, consistent synchrony is independent of the intrinsic properties of individual neurons. This is a likely consequence of synchrony driven by the strong network inter-connectivity and PING-like gating (i.e., suffiently strong bursts of inhibitory activity suppress firing in both excitatory and inhibitory populations) [17], intuitively explaining the similar dynamics arising from both dynamic and tonic changes to $g_{k_{s}}$. Meanwhile, the divergent behavior with dominant intra-connectivity networks is expected since these networks’ ability to synchronize is dependent upon cellular properties at high $g_{k_{s}}$ [17], motivating a more rigorous analysis of their behavior.

### Desynchronization in dominant intra-connectivity networks is sensitive to the rate of cholinergic modulation

To verify our intuition that only dominant intra-connectivity networks respond differentially to dynamic cholinergic modulation, we conducted simulations with varying rates of $g_{k_{s}}$ downregulation. Calculating the Synchrony Measure of the excitatory population (see Materials and Methods), we compared firing patterns between tonic and dynamic models across the entire $g_{k_{s}}$ range (Fig 4). For the dynamically modulated networks, we compute the Synchrony Measure across moving time windows, allowing us to visualize the temporal evolution of synchrony as $g_{k_{s}}$ declines.

Validating our predictions, the degree of network synchrony in dominant inter-connectivity networks is similar under both tonic and dynamic modulation and largely indifferent to the rate of modulation (Fig 4A). However, dominant intra-connectivity networks are consistently more synchronized at a given $g_{k_{s}}$ arrived at dynamically rather than set tonically (Fig 4B and 4C). In dynamically modulated networks, for high $g_{k_{s}}$ values, excitatory synchrony as quantified by the Synchrony Measure is largely independent of the rate at which $g_{k_{s}}$ declines. However, as $g_{k_{s}}$ further decreases, the system becomes less synchronous (reflected in a decreasing Synchrony Measure) in a manner sensitive to the rate of modulation. This is quantified in Table 1: slower rates of modulation consistently lead to desynchronization (Synchrony Measures < 0.2; this approximate cut off value has been used in previous literature [47] and is qualitatively associated with a conspicuous lack of network synchrony in raster plots) at smaller $g_{k_{s}}$ values and over longer time periods. The magnitude of the synchrony difference between dynamically and tonically modulated networks diminishes as $g_{k_{s}}$ decreases until the network is completely asynchronous (Synchrony Measure of approximately 0). These patterns are broadly present with (Fig 4B) and without (Fig 4C) cholinergic modulation of the inhibitory cells, with the rate of modulation affecting the $g_{k_{s}}$ value at which both types of networks desynchronize (see Table 1). However, synchronous firing lasts longer without cholinergic modulation of the inhibitory cells. This follows from the inhibitory cells being more excitable and capable of forming tighter bursts with the absence of the m-current, which improves the consistency of inhibitory signaling to the excitatory population.

**Table 1:** Time to desynchronize for dominant intra-connectivity networks with different rates of $g_{k_{s}}$ modulation. Desynchronization is quantified as occurring at the midpoint of the time window at which the Synchrony Measure first is below 0.2 (see dotted line in Fig 4).

Rate of $g_{k_{s}}$ decline	Time to desynchronize	$g_{k_{s}}$ at desynchronization
1.5 (mS/cm^2^)/s	475 ms	0.78 mS/cm^2^
0.75 (mS/cm^2^)/s	850 ms	0.86 mS/cm^2^
0.38 (mS/cm^2^)/s	1500 ms	0.94 mS/cm^2^
0.19 (mS/cm^2^)/s	2760 ms	0.98 mS/cm^2^
1.5 (mS/cm^2^)/s, only E cells	605 ms	0.59 mS/cm^2^
0.75 (mS/cm^2^)/s, only E cells	1170 ms	0.62 mS/cm^2^
0.38 (mS/cm^2^)/s, only E cells	2460 ms	0.58 mS/cm^2^
0.19 (mS/cm^2^)/s, only E cells	5080 ms	0.55 mS/cm^2^

The comparisons between the dynamic and tonic networks as well as the similar dynamics exhibited at high $g_{k_{s}}$ for all rates of modulation demonstrate that past firing patterns play a potentially outsized role in network dynamics during dynamic cholinergic modulation. The precipitous transition from synchronous to asynchronous dynamics as $g_{k_{s}}$ declines suggests that there may be a “critical” concentration of ACh that is necessary to initiate desynchronization and that network behavior matches predictions from tonically modulated networks past this point. These analyses confirm that only networks with dominant intra-connectivity, when subject to dynamic cholinergic modulation, diverge from the dynamics of analogous tonically modulated systems. Their synchronization properties cannot be encapsulated as a function of the ACh concentrations at a given time alone, but rather depend upon the temporal evolution of ACh concentration—and in particular how that temporal evolution influences the history of firing activity. In the following, we continue study of networks with strong intra-connectivity using an exemplar $g_{k_{s}}$ downregulation rate of 0.67 mS/cm^2^, which we found to be slow enough to allow for gradual changes in firing patterns while still remaining computationally tractable.

### The mechanism of ACh-driven desynchronization is especially sensitive to changes in E-I connectivity strength

With our focus on networks with strong intra-connectivity appropriately motivated, we further delineated the potentially interacting effects of dynamic cholinergic modulation and synaptic strengths on oscillatory activity. We independently varied E-I and I-E synaptic strengths to more precisely establish their effects on the dynamics described above. For both inter-connectivity synapses we quantify network dynamics as their weights were increased up to and including the value used in “dominant inter-connectivity” networks. This was done in networks with and without cholinergic modulation of the inhibitory neurons (see Discussion [48]).

**Fig 5:**
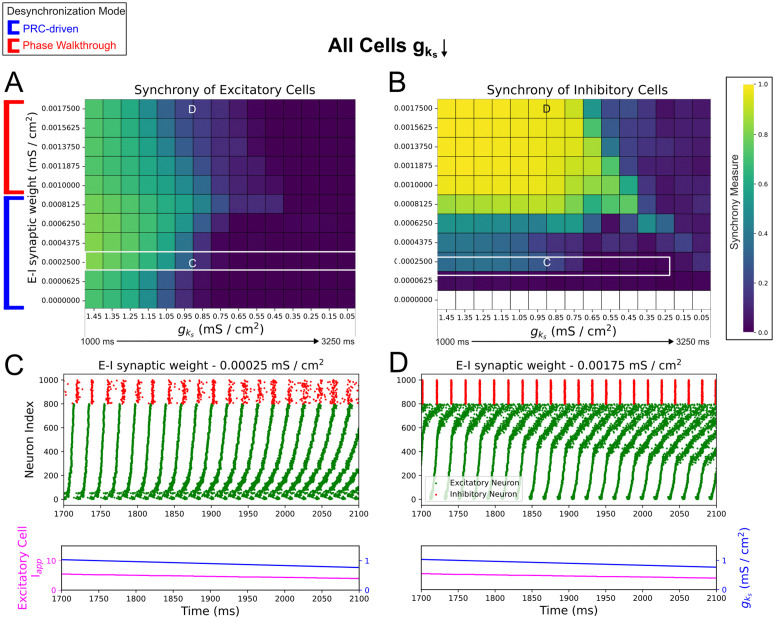
E-I connectivity controls speed and mechanism of ACh-induced desynchronization in dominant intra-connectivity networks. **A-B**: Heatmaps of excitatory (Panel **A**) and inhibitory (Panel **B**) synchrony averaged over 10 independent simulations in networks with varying E-I connectivity strength. Cholinergic modulation is implemented with $g_{k_{s}}$ decline rate 0.67 (mS/cm^2^)/s, carried out for all cells. E-E, I-I, and I-E synaptic weights are fixed at 0.000125 mS/cm^2^, 0.0005 mS/cm^2^, and 0.00025 mS/cm^2^ respectively, with the default dominant intra-connectivity networks denoted with special white / black borders. **C-D**: Raster plots showing 400 ms periods of activity selected from networks of differing synaptic weights as labeled. Variations in E-I connectivity shift the duration of synchronous firing that are underscored by changes in the mode of desynchronization (red/blue labels to the left of Panel **A**). Panel **C** and **D** showcase how these distinct modes of desynchronization manifest: PRC-driven desynchronization occurs due to the attenuation of the phase delay region in the excitatory cells’ PRC and manifests first in desynchronization of neurons with the highest external inputs (Panel **C**), while phase walkthrough-driven desynchronization occurs due to the inhibitory bursts arriving progressively earlier and disrupting the firing times of excitatory cells with the lowest external inputs (Panel **D**).

**Fig 6:**
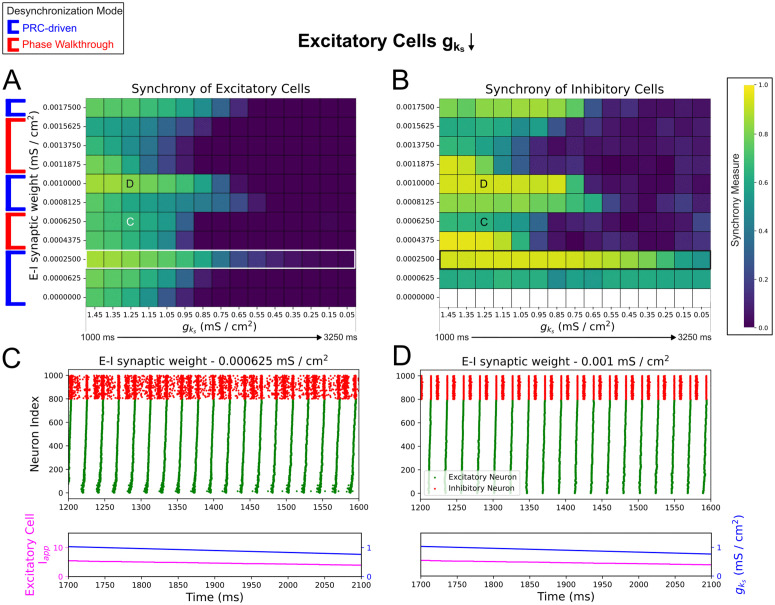
With cholinergic modulation restricted to excitatory neurons, varying E–I connectivity in dominant intra-connectivity networks allows the mechanism of desynchronization to repeatedly alternate. **A-B**: Heatmaps of excitatory (Panel **A**) and inhibitory (Panel **B**) synchrony averaged over 10 independent simulations in networks with varying E-I connectivity. Cholinergic modulation is implemented with $g_{k_{s}}$ decline rate 0.67 (mS/cm^2^)/s and is carried out for only excitatory cells while inhibitory cells’ $g_{k_{s}}$ is permanently set to 0 (mS/cm^2^)/s. E-E, I-I, and I-E synaptic weights are fixed at 0.000125 mS/cm^2^, 0.0005 mS/cm^2^, and 0.00025 mS/cm^2^ respectively, with the default dominant intra-connectivity networks denoted with special white / black borders. **C-D**: Raster plots showing 400 ms periods of activity selected from networks of differing synaptic weights as labeled. The desynchronization mode of the network alternates between the PRC and phase walkthrough regimes as E-I connectivity increases (Panels **A** and **B**). Panels **C** and **D** demonstrate the development of multiple inhibitory population bursts per excitatory population burst, with more organized inhibitory bursts corresponding to systems in which synchronous activity persists for lower $g_{k_{s}}$ values.

Interestingly, there is a non-monotonic relationship between E-I connectivity strength and the duration of network synchrony in these systems, both when cholinergic modulation affects both excitatory and inhibitory (Fig 5) and just excitatory (Fig 6) neurons. In the former case (Fig 5A), increases to the E-I synaptic weight up to 0.0008125 mS/cm^2^ delay desynchronization in the network. Increases above this threshold instead hasten desynchronization, causing it to occur at higher $g_{k_{s}}$ values. As illustrated by exemplar raster plots (Fig 5C and 5D), we found that desynchronization in networks with an E-I synaptic weight below this threshold—which includes the default dominant intra-connectivity network studied in previous sections—starts with cells with higher external inputs (Fig 5C). Meanwhile, above this threshold, we observe a distinct pattern of desynchronization that starts with cells with lower external inputs (Fig 5D). We hypothesized that these dichotomous patterns were indicative of two distinct mechanisms of ACh-modulated desynchronization, demarcating two regimes (red and blue boundaries in Fig 5A) determined by the strength of E-I connectivity. We note that the identification of this distinction relies largely on our instantiation of heterogeneous tonic external inputs to the excitatory population.

The pattern of desynchronization below the threshold E-I synaptic weight is, as for our dominant intra-connectivity network, viably explained by the changes to the excitatory cells’ PRC properties induced by $g_{k_{s}}$ decline and is thus termed “PRC-driven.” Although all the networks in this range desynchronize in this manner, the increase in E-I connectivity delays desynchronization by better entraining the inhibitory population. This is evidenced in the qualitative relationship between the magnitude of the initial synchrony in the inhibitory population (leftmost column of Fig 5B) and the duration of excitatory synchronous activity (Fig 5A). This occurs as more synchronous inhibitory bursts create a stronger and more uniform inhibitory signal to each of the excitatory cells, minimizing the cell-to-cell variability in this signal and strengthening any resulting gating effects.

Thus, in spite of weak I-E synapses, desynchronization is delayed thanks to gating effects. However, unlike with PING-driven networks, the inhibitory signaling here only preserves existing synchronous activity rather than serving as the impetus for its formation. This is confirmed when we set the strength of the E-I synaptic weight to 0 mS/cm^2^: while inhibitory cells are inactive (white bottom row of Fig 5B), we find that the excitatory cells’ synchrony shows no apparent difference from the lowest non-zero E-I synaptic weight (network with E-I synaptic weight 0.0000625 mS/cm^2^ in Fig 5A).

We attribute the phenomenon in the second regime (red-bounded networks in Fig 5A), where desynchronization begins with cells with the lowest external inputs, to “phase walkthrough.” This terminology, borrowed from previous PING literature [28], refers to a burst of inhibitory activity that occurs before the spiking of the less driven excitatory cells, but not early enough to suppress said spiking. Raster plot inspection revealed that once a sufficiently low $g_{k_{s}}$ value is reached in these networks, high E-I connectivity leads inhibitory bursts to arrive progressively earlier relative to the excitatory bursts, breaking the phase-lock between the excitatory and inhibitory populations—the defining characteristic of phase walkthrough. When the inhibitory bursts arrive early enough that they precede and disrupt the spiking of the excitatory cells with the lowest external inputs, signaling between the E and I populations becomes weaker with more cell-to-cell variability, initiating desynchronization of the excitatory population.

When cholinergic modulation is restricted to excitatory cells (Fig 6A and 6B) there are multiple points at which increasing E-I synaptic strength shifts between increasing and decreasing the duration of synchronous activity. Correspondingly, we note a set of “optimal” E-I synaptic weights (0.00025, 0.0008125, 0.001, 0.00175 mS/cm^2^ in Fig 6A) at which relatively long-lasting synchronous activity is present. By qualitatively classifying each system as desynchronizing by either a PRC-driven or phase walkthrough mechanism—determining whether desynchronization begins with cells with the highest or lowest external inputs, via raster plot inspection, as described above—we determined that these E-I synaptic weights lie at points of transition between PRC-driven and phase walkthrough-driven desynchronization regimes.

We explain this behavior through the higher excitability of the inhibitory cells locked at $g_{k_{s}}=0$ mS/cm^2^. This excitability allows each inhibitory cell to respond with multiple spikes in response to each excitatory burst as E-I connectivity is increased (Fig 6C and 6D); notably, this phenomenon was never observed in the scenario described in Fig 5. However, the number and timing of responses from each inhibitory cell does not change in a uniform manner: for example, for an E-I synaptic weight of 0.000625 mS/cm^2^ (Fig 6C), the inhibitory clusters that follow each excitatory burst are highly temporally disorganized, but for 0.001 mS/cm^2^ we observe two well-defined, tight bursts in response to each excitatory burst (Fig 6D). This general trend is quantified in the leftmost column of Fig 6B, where we observe a rough correspondence between that the initial synchrony of the inhibitory population and E-I synaptic weights that promote longer lasting synchronous dynamics.

The aforementioned optimal E-I synaptic weights occur when the inhibitory cells are able to organize into well-defined, tight bursts and more effectively delay desynchronization through gating signals. For example, at 0.00025 mS/cm^2^, the initial inhibitory synchrony is high (leftmost column of Fig 6B), and the excitatory cells are able to stay synchronized for a relatively longer duration (Fig 6A). Further increase in the E-I synaptic weight to 0.0004375 mS/cm^2^ results in earlier desynchronization through phase walkthrough, despite high initial inhibitory synchrony, as was also the case with the phase walkthrough regime in Fig 5A and 5B. As E-I connectivity is scaled past an E-I synaptic weight at which phase walkthrough occurs, the increase in the inhibitory cells’ firing frequency, coupled with the strong I-I connectivity slowing the inhibitory bursts, appears to allow a return to the regime where network desynchronization is driven by PRC effects and phase walkthrough does not occur (for example, 0.0008125 mS/cm^2^ case in Fig 6A and 6B).

Thus, E-I connectivity plays an important role in controlling the manner and rate of desynchronization by controlling the timing and strength of the inhibitory signaling. Subtle differences in this connectivity may drastically alter how a network responds to cholinergic modulation, showing that network connectivity works together with ACh-induced changes in cellular properties to determine network dynamics.

**Fig 7:**
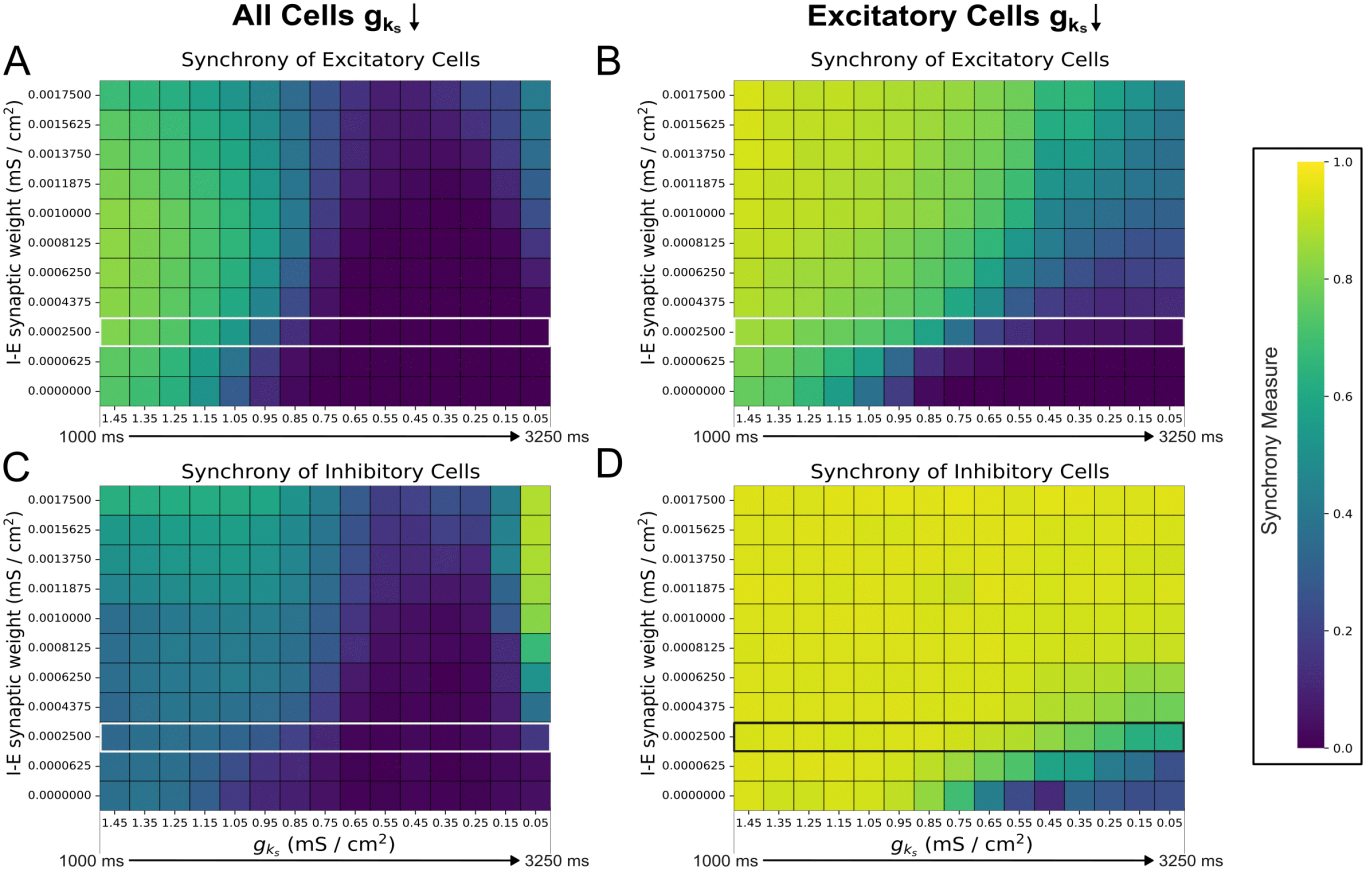
I-E connectivity prolongs synchronous firing for dominant intra-connectivity networks subjected to cholinergic modulation. **A-D**: Heatmaps of excitatory (Panels **A** and **C**) and inhibitory (Panels **B** and **D**) synchrony averaged over 10 independent simulations in networks with varying I-E connectivity. Cholinergic modulation is implemented with $g_{k_{s}}$ decline rate 0.67 (mS/cm^2^)/s, carried out for all cells (Panels **A** and **B**) or for only excitatory cells while inhibitory cells’ $g_{k_{s}}$ is permanently set to 0 mS/cm^2^ (Panels **C** and **D**). E-E, I-I, and E-I synaptic weights are fixed at 0.000125 mS/cm^2^, 0.0005 mS/cm^2^, and 0.00025 mS/cm^2^ respectively, with the default dominant intra-connectivity networks denoted with special white / black borders. Strengthening I-E connectivity gradually increases the duration of network synchrony (Panels **A** and **C**) and enables the reemergence of synchrony at low $g_{k_{s}}$ (Panel **A**).

In contrast, strengthening I-E connectivity uniformly delays PRC-driven desynchronization (Fig 7A and Fig 7C) with no cases of phase walkthrough, as verified by inspecting the raster plots and finding that desynchronization consistently began with the cells with a larger tonic external input. The delay in PRC-driven desynchronization here may be attributed to the increased I-E synaptic weights strengthening inhibitory signaling to the excitatory cells and consequently strengthening gating effects; as was noted above for the PRC-driven desynchronization regime of Figs 5A and 6A, this gating has some similarities to the PING mechanism.

Notably, in Fig 7A, weakly synchronous activity (Synchrony Measure values of approximately 0.4 at I-E synaptic weight 0.001375 mS/cm^2^ and above) reemerges after desynchronization for sufficiently high I-E connectivity. This resurgence occurs at low $g_{k_{s}}$ values, where the inhibitory cells are more excitable and better able to reorganize into synchronous bursts (Fig 7B), allowing them to re-initiate oscillatory network activity. Conversely, when cholinergic modulation is restricted to the excitatory cells and inhibitory cells’ $g_{ks}$ is locked at 0 mS/cm^2^ (Fig 7C and 7D), highly synchronized inhibitory bursts are consistent through time for most I-E synaptic weights, preventing the excitatory cells from desynchronizing at moderate values of $g_{k_{s}}$.

**Fig 8:**
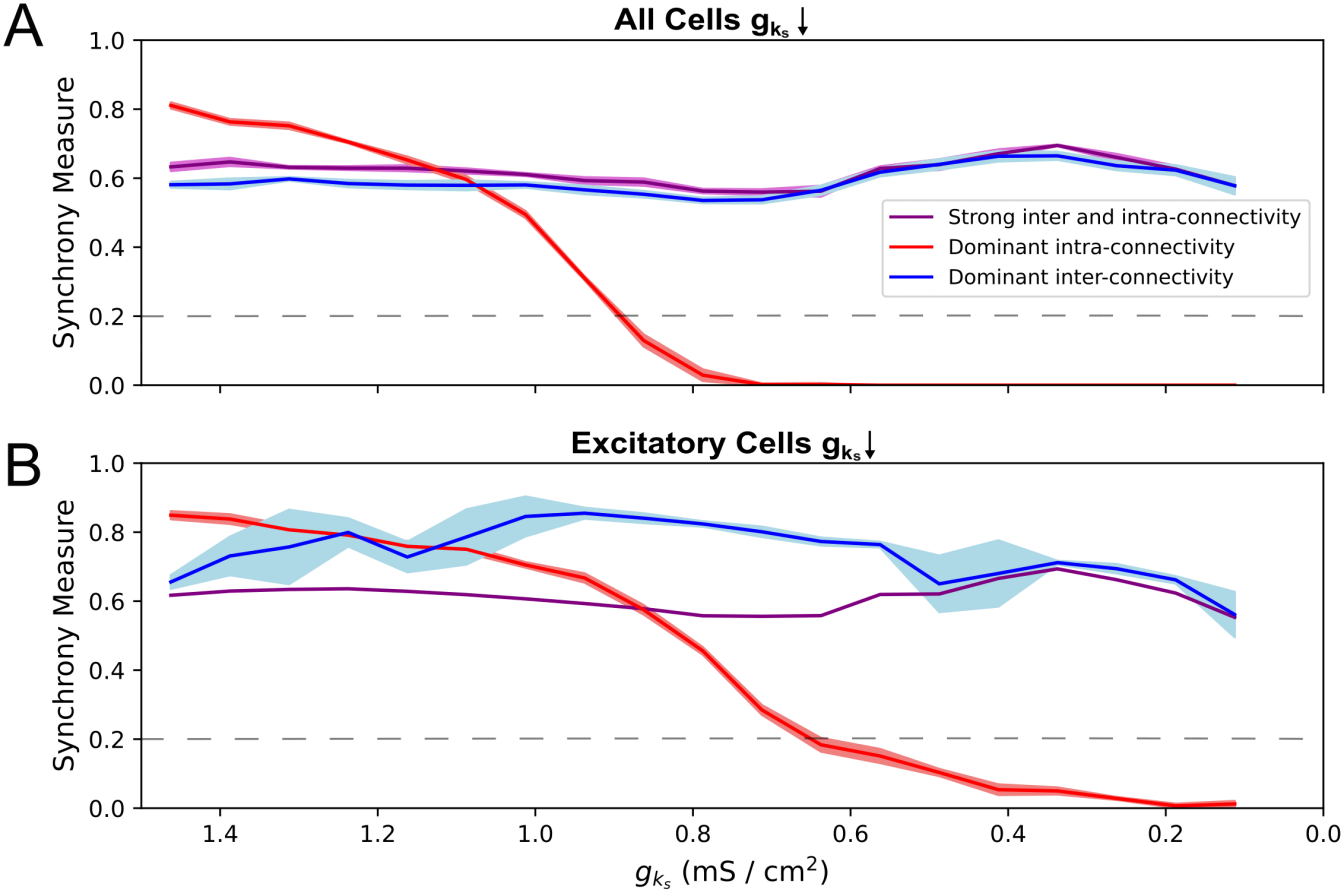
In systems with both strong inter- and intra-connectivity, network response is dominated by interconnectivity-driven effects. **A-B**: Synchrony measure of excitatory cells plotted over $g_{k_{s}}$ for networks with varying network topologies and cholinergic modulation for all cells (Panel **A**) or for only excitatory cells (Panel **B**). Synaptic weight parameters for dominant intra-conectivity and dominant inter-connectivity synaptic weights are as specified in the Materials and Methods and as follows for strong inter and intra-connectivity networks: E-I, I-E = 0.00175, E-E = 0.000125, I-I = 0.0005 mS/cm^2^. All measures are the mean based on 10 independent simulations, with ± standard deviation shading displayed. Network dynamics are classified as desynchronized when the Synchrony Measure is below 0.2 (grey-dashed lines in each panel). When E-I and I-E synaptic weights are both high, network behavior is largely uninfluenced by cholinergic modulation, as predicted by strong inter-connectivity.

Thus, while both E-I and I-E synapses mediate ACh-driven desynchronization, changes in E-I connectivity exert stronger control over the desynchronization rate while changes in the I-E connectivity have a weaker and more uniform effect. Previous research [17] suggests that the PING-driven effects of strong inter-connectivity would dominate the ACh-driven effects of strong intra-connectivity when all synaptic weights are strong. We validate this in Fig 8: in networks with simultaneously strong intra- and inter-connectivity (purple networks in Fig 8A and 8B in which each synaptic weight matches the value in the associated “dominant” connectivity network), the stability created by strong inter-connectivity promotes activity qualitatively matching that of dominant inter-connectivity networks (blue networks in Fig 8A and 8B). Indeed, despite some quantitative differences and fluctuations in the Synchrony Measure, these values remain well within the range indicative of synchronous network activity for all $g_{k_{s}}$ values.

## Discussion

**Fig 9:**
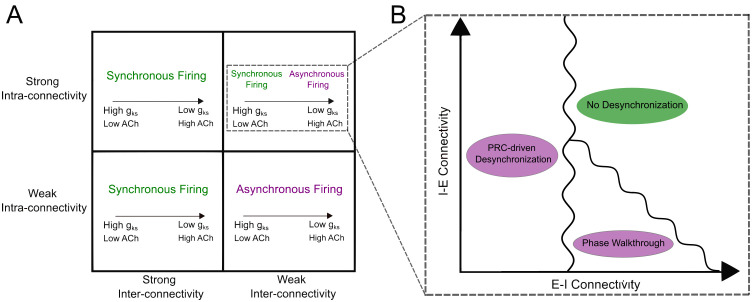
Summary figure depicting the network topologies where dynamic cholinergic modulation desynchronizes activity, along with the underlying mechanisms of desynchronization. **A**: Dynamic cholinergic modulation alters firing patterns and desynchronizes activity only in networks where intra-connectivity dominates over inter-connectivity. Under these conditions, the dynamics of the network are dependent on the timescale of cholinergic modulation. **B**: For networks with strong intra-connectivity, desynchronization can occur in two different modes, one mediated by PRC-driven effects and the other by phase walkthrough, with the latter arising when E-I synapses are sufficiently strong.

By studying a computational neuronal network subject to dynamic cholinergic modulation of the m-current, we here delineate how time-varying ACh concentrations and network connectivity interact to control synchronous network activity in *in silico* cortical microcircuits. It has been previously established [17] that E-I networks with dominant intra-connectivity are strongly affected by changing cholinergic tone, with oscillatory behavior at low ACh and desynchronized firing at high ACh (Fig 9A). Here, we demonstrate that how these networks desynchronize is sensitive to how ACh concentrations change over time, a factor omitted in canonical computational models considering only a set cholinergic tone. The uniqueness of the network dynamics in response to time-varying ACh levels motivated our introduction of small changes to E-I and I-E connectivity strengths (Fig 9B), which identified that E-I synapses have a surprisingly disproportionate and non-monotonic influence over the mechanism and timing of network desynchronization. This can be attributed to two distinct mechanisms of action, one driven by phase walkthrough effects and another driven by PRC properties of the excitatory cells.

Experimental evidence strongly suggests that ACh acts on sub-second timescales via muscarinic receptors [5,12] as phenomenologically approximated in this work. Indeed, the primary source of cholinergic projections to the cortex, the basal forebrain, delivers precisely timed cholinergic transients [6,49], promoting a transition from low-frequency rhythmic activity to desynchronized firing as seen in local field potentials and EEG recordings [3,50]. Such ACh-induced desynchronization arises not only during sleep-wake cycles, but also rapidly during tasks that engage sensory and motor skills [51,52]. VNS also promotes rapid desynchronization of EEG activity [13] in a manner likely dependent, at least in part, on muscarinic activity [14].

Parallel lines of inquiry have specifically implicated cholinergic modulation of the m-current in desynchronizing neuronal activity in the pedunculopontine nucleus [53,54] and during sleep-wake transitions in the cortex [55,56]. Additional electrophysiological studies in the hippocampus reveal that Kv7/m-channels regulate network synchronization [57–59], with these channels modulating gamma-band oscillations and suppressing intrinsic bursting. Our work is a pivotal *in silico* step towards mechanistic explanations for these observations.

We find that dynamically increasing cholinergic tone causes rapid desynchronization in networks where oscillations are driven by intrinsic cellular properties dependent upon m-current activity [45], but not in networks with oscillations arising from PING-like gating mechanisms [28,60]. This desynchronization is driven by dynamically changing PRC properties of excitatory cells and/or excessive excitation of inhibitory cells causing phase walkthrough [28]. Viewed in the context of the experimental observations described above, our study provides *in silico* confirmation that sub-second variations in cholinergic tone exert crucial control over microcircuit oscillations and makes additional predictions regarding the synaptic strengths of microcircuits most affected in this manner.

This study purposefully focuses on cholinergic modulation via muscarinic receptors to facilitate direct comparison with the extensive computational literature focused on static modulation of the muscarinic receptor-gated m-channel [29–33]. This choice allows us to leverage this existing knowledge to propose mechanisms explaining the new phenomena observed in our model microcircuits. Furthermore, this choice respects the mounting experimental evidence that muscarinic receptors play a crucial role in desynchronizing cortical activity [12,50] and in regulating cognitive functions such as attentional modulation [61] over sub-second timescales.

Our approach is further motivated by the contemporary study of VNS, which suggests its therapeutic effects rely on cholinergic pathways [62,63] and are dependent upon muscarinic activity [14]. While the mechanisms of action of VNS are not yet fully understood [62–64], its efficacy in post-stroke motor rehabilitation [62,65–67] interestingly requires stimulation precisely timed with a successful motor task. In related learning tasks, VNS-driven learning improvements require cholinergic activity [68]. A viable interpretation of these findings, along with results showing VNS rapidly desynchronizes cortical EEG signals [13], is that a cholinergic signal may encode the sub-second timing of a VNS input by altering cortical oscillations. Our novel *in silico* findings propose the first viable mechanism of action by which this might occur and therefore represent additional support for a “special” role for ACh in VNS’s therapeutic effects. Building on our results, *in silico* approaches are well suited to predict the temporal profile of the cholinergic response to VNS and test its influence over cortical microcircuits, generating predictions that can be tested in future studies of EEG activity in patients undergoing VNS-paired therapy.

While our study focuses on the disruption of cortical synchrony (and associated oscillatory activity) by increased ACh, in some experimental settings cholinergic transients have a synchronizing effect—for instance, in the formation of gamma rhythms in the prefrontal cortex during cue-detection [69]. This phenomenon has been captured computationally in E-I networks with pulsatile time-varying modulation of the m-current [33]. The distinct experimental phenomena captured by our model and the work of [33] is reflected in an important *in silico* nuance: [33] does not control for increases in neuronal baseline firing frequency induced by the effect of $g_{k_{s}}$ modulation on the F-I curve (as done in our work) given the impermanent nature of their modeled ACh increases. This implies that such synchronization effects are likely more dependent on the heightened excitability induced by F-I curve modulation, as suggested in [33] itself, than the PRC modulation effects of primary focus in our study. This not only reconciles these seemingly disparate findings regarding cholinergic modulation’s influence on network synchrony, but highlights the need for *in silico* study to parse out the most salient effects of cholinergic modulation on network dynamics in varying settings (potentially reflecting the presence of other neuromodulators influencing neuronal excitability).

We examined networks with and without an active m-current in inhibitory cells in consideration of the conflicting evidence regarding muscarinic receptor prominence among parvalbumin interneurons in the visual cortex [48]. We found that networks with cholinergic modulation for inhibitory cells may allow for partially synchronous firing in high ACh networks, a result predicted by previous study of the m-channel’s influence on patterning in purely inhibitory networks [15]. Interestingly, both PRC-driven and phase walkthrough-driven desynchronization modes we defined arose in networks with exclusive cholinergic modulation of excitatory cells. Notably, this conclusion requires observing the change in firing patterns over time, therefore requiring the model of dynamic cholinergic modulation implemented here. By testing systems in which ACh both does and does not affect inhibitory cells, we showcase that these relationships between dynamic cholinergic modulation and the strength of synaptic connectivity—namely, that the effects cholinergic modulation at sub-second timescales are strongly dependent upon network connectivity—are at minimum partially generalizable to systems with various inhibitory cell types.

As is the case in all computational studies, the results of this work must be interpreted relative to the limitations imposed by our modeling choices [70]. In order to best facilitate comparison with existing computational studies [17,29–33], we exclusively consider localized microcircuit dynamics on the order of a few seconds where cholinergic signaling is assumed to be spatiotemporally uniform. The highly distributed nature of cholinergic projections to the cortex [5,71] and the diversity of cholinergic receptors at different sites [3] are ripe topics for anatomically-detailed future study. We focus here on modulation of a model m-current shown previously [19,24] to reproduce the effects of M1-receptor mediated cholinergic signaling on excitability and PRC properties of cortical pyramidal neurons [11]. Accounting for the effects of nicotinic receptors and alternative muscarinic receptor subtypes (M1-M5) would interfere with our ability to isolate PRC changes accompanying cholinergic modulation and in turn articulate mechanistic explanations of our observed dynamics. Whether these mechanisms influence the dynamics of more detailed models—accounting not only for these other cholinergic receptors, but secondary effects of ACh on presynaptic glutamate and GABA release [72]—is another fascinating topic for future study. While we have therefore simplified the complexity of cholinergic signaling in this system, this approach follows a long tradition in computational neuroscience of approaching complex new questions by first deriving foundational theory in rationally simplified settings. Here, our findings—that the timecourse of cholinergic modulation is a crucial mediator of synchrony in cortical microcircuits—should motivate both the inclusion of time-varying cholinergic signals in future computational studies and continued experimental characterization of these signals with sub-second resolution *in vivo*.
